# Oilseed rape (*Brassica napus*) as a resource for farmland insect pollinators: quantifying floral traits in conventional varieties and breeding systems

**DOI:** 10.1111/gcbb.12438

**Published:** 2017-03-10

**Authors:** Jonathan M. Carruthers, Samantha M. Cook, Geraldine A. Wright, Juliet L. Osborne, Suzanne J. Clark, Jennifer L. Swain, Alison J. Haughton

**Affiliations:** ^1^ Rothamsted Research West Common Harpenden Hertfordshire AL5 2JQ UK; ^2^ Royal Society of Biology Charles Darwin House, 12 Roger Street London WC1N 2JU UK; ^3^ Centre for Behaviour and Evolution Institute of Neuroscience Newcastle University Newcastle upon Tyne NE1 7RU UK; ^4^ Environment and Sustainability Institute University of Exeter Penryn Campus Penryn Cornwall TR10 9FE UK

**Keywords:** canola, cytoplasmic male sterility, floral traits, genic male sterility, hybrids, nectar, open‐pollinated, pollinator declines, rapeseed, sustainable intensification

## Abstract

Oilseed rape (OSR;* Brassica napus* L.) is a major crop in temperate regions and provides an important source of nutrition to many of the yield‐enhancing insect flower visitors that consume floral nectar. The manipulation of mechanisms that control various crop plant traits for the benefit of pollinators has been suggested in the bid to increase food security, but little is known about inherent floral trait expression in contemporary OSR varieties or the breeding systems used in OSR breeding programmes. We studied a range of floral traits in glasshouse‐grown, certified conventional varieties of winter OSR to test for variation among and within breeding systems. We measured 24‐h nectar secretion rate, amount, concentration and ratio of nectar sugars per flower, and sizes and number of flowers produced per plant from 24 varieties of OSR representing open‐pollinated (OP), genic male sterility (GMS) hybrid and cytoplasmic male sterility (CMS) hybrid breeding systems. Sugar concentration was consistent among and within the breeding systems; however, GMS hybrids produced more nectar and more sugar per flower than CMS hybrid or OP varieties. With the exception of ratio of fructose/glucose in OP varieties, we found that nectar traits were consistent within all the breeding systems. When scaled, GMS hybrids produced 1.73 times more nectar resource per plant than OP varieties. Nectar production and amount of nectar sugar in OSR plants were independent of number and size of flowers. Our data show that floral traits of glasshouse‐grown OSR differed among breeding systems, suggesting that manipulation and enhancement of nectar rewards for insect flower visitors, including pollinators, could be included in future OSR breeding programmes.

## Introduction

*Brassica napus* (oilseed rape, OSR) is the world's third largest source of vegetable oil (USDA, 2016a), supplying the food and feed industries, and continues to be a key biofuel feedstock (USDA, 2016b). While OSR is capable of self‐pollination, insect visitation to its flowers is important, as it enhances crop value through increased yield and quality (Morandin & Winston, 2006; Bommarco *et al*., 2012; Bartomeus *et al*., 2014; Hudewenz *et al*., 2014). However, pollination services in farmed landscapes are threatened, and global declines in insect pollinator abundance and richness (Biesmeijer *et al*., 2006; Potts *et al*., 2010; Cameron *et al*., 2011; Carvalheiro *et al*., 2013) have been attributed, in part, to limited quality and availability of food resource, particularly as a result of land use change associated with agricultural intensification (Klein *et al*., 2007; Potts *et al*., 2010; Goulson *et al*., 2015). Recent evidence has revealed the relative paucity of nectar sources in arable farmland compared with seminatural habitats (Baude *et al*., 2016) and in such landscapes where alternative food sources are limited, mass‐flowering crops, such as OSR, to create large spatio‐temporal pulses of nectar and pollen that are exploited by wild and managed insect pollinators (Stanley & Stout, 2013; Gill & O'Neal, 2015; Requier *et al*., 2015). Cultivation of OSR has been shown to enhance within‐season pollinator abundance (Westphal *et al*., 2003; Williams *et al*., 2012) and more significantly, between‐year populations (Jauker *et al*., 2012; Holzschuh *et al*., 2013; Riedinger *et al*., 2015).

Wild plants have evolved strategies to maximize genetic diversity and reproduction potential, where floral trait selection, such as nectar production, has led to c. 87% plant species being pollinated by animals (Ollerton *et al*., 2011). Flowers use floral nectar as a reward for animal (anthophilous) pollinators, and it is an important, nutrient‐rich dietary resource for many flower visitors, e.g. Lepidoptera (Jervis & Boggs, 2005; Lebeau *et al*., 2016). The composition of nectar has evolved to attract pollinators and plant defenders and to protect against nectar robbers and pathogens (Kessler *et al*., 2008; Heil, 2011; Nicolson *et al*., 2015), and although the precise composition of floral nectar varies within and between species (Burkle & Irwin, 2009; Baude *et al*., 2011), it generally comprises up to 80% w/w sugars (sucrose, glucose and fructose), with the remainder comprising amino acids and lipids, as well as complexes of secondary metabolites (Baker & Baker, 1983; Nicolson & Thornburg, 2007). While pollination ecology studies of the interactions between pollinators and nectar are well established, e.g. Goulson (1999), Schaefer *et al*. (2004), Mayer *et al*. (2011), those elucidating the molecular mechanisms behind floral nectar production have only recently made progress and have demonstrated that production, synthesis, secretion and regulation are under genetic and hormonal control (Radhika *et al*., 2010; Ruhlmann *et al*., 2010; Liu & Thornburg, 2012; Bender *et al*., 2013; Lin *et al*., 2014; Wang *et al*., 2014), leading to suggestions that manipulating these mechanisms in crop plant‐breeding programmes could increase the potential value of crops to insect pollinators in the context of improving food security through sustainable intensification (Bailes *et al*., 2015).

Oilseed rape is the focus of intensive, commercial breeding programmes that culminate in the registration of new, certified varieties that assure minimum standards of phenological and morphological metrics of yield and disease and quality (OECD, 2016). However, the value to insect visitors and pollinators of new varieties of flowering crops, such as OSR, is not considered in breeding programmes or current certification criteria (OECD, 2016). Non‐GM, conventionally bred OSR varieties are developed either through classical line‐breeding methods, making crosses and selecting the most promising genotypes to produce uniform, open‐pollinated (OP) varieties (Friedt & Snowdon, 2009), or as hybrids that demonstrate improved yields through heterosis (Frauen *et al*., 2003; Rai *et al*., 2007). Hybrid seed is obtained from a male‐sterile parent line through crossing with a male‐fertile parent line that confers fertility restorer genes to the F1 offspring, which then produce pollen and seed. To increase yield potential further, male‐sterile plants are created by using genes located in either the cytoplasmic or nuclear genome that induce male sterility (Delourme & Budar, 1999). In lines with cytoplasmic male sterility (CMS), a mutation in the mitochondrial genome inhibits the development of pollen, whereas in lines with genic male sterility (GMS), male sterility develops due to the action of genes located in the nucleus (Ke *et al*., 2005). Hybrid and OP varieties are cultivated in Europe, whereas hybrids, together with genetically modified, varieties are grown in North America (Friedt & Snowdon, 2009) and Australia (Oliver *et al*., 2016).

Oilseed rape breeding programmes have conserved the function of nectar production in varieties (Pernal & Currie, 1998; Pierre *et al*., 1999), but little is known about the production of nectar in the three breeding systems. There is limited evidence that Ogura cytoplasm used in CMS hybrids may result in less developed nectaries with associated lowered nectar production (Pelletier *et al*., 1987; Mesquida *et al*., 1991), but Pierre *et al*. (1999) found no differences between the nectar volumes and sugar concentrations between three Ogura CMS hybrids and three OP varieties. Nectar secretion of GMS hybrids has not been compared with CMS hybrids or OP varieties.

The total resource for insect visitors provided by an OSR plant is a function of the nutritional composition of nectar and pollen supplied by each flower and the total number of flowers produced per plant. Additionally, the accessibility and composition of nectar rewards may compromise the utility of flowers to some insect species. For example, beekeepers have noted concern that varieties of OSR with high glucose nectars result in crystallized honey stores in the hive that cannot easily be exploited and used by bees in the colony and produce rapidly granulating, lower value honeys (Calder, 1986). The growth, development and function of floral characters may be resource limited in plants (Diggle, 1997; Galen, 1999); indeed, *B*. *napus* plants, which exhibit an indeterminate growth habit (Wang *et al*., 2009), adjust the number, but not the size, of flowers they produce according to planting density (Cresswell *et al*., 2001) and visitation by insects (Mesquida *et al*., 1988b). However, there may be additional, inherent constraints on the number and size of flowers due to variety or breeding system. Insect plant visitors use floral characters, such as flower size (Conner & Rush, 1996; Makino *et al*., 2007), as visual signals of resource availability, but there is conflicting evidence from the closely related *B*. *rapa* that flower size may be an honest indicator of nectar status (Davis *et al*., 1996; Knauer & Schiestl, 2015).

By quantifying and elucidating differences in the inherent floral traits of winter OSR varieties and breeding systems, it could be possible to work towards breeding and cultivating OSR with pollinator‐positive traits to support an important ecosystem service in the production of rapeseed oil. Thus, we quantified a range of inherent floral traits of glasshouse‐grown, contemporary certified varieties of OSR, representing three conventional breeding systems. Specifically, we quantified nectar volume and its sugar mass and composition produced per flower over a 24‐h period, a surrogate measure of flower size and nectar production per plant per 24 h in OSR varieties from OP, CMS hybrid and GMS hybrid breeding systems.

## Materials and methods

### Plant material

Twenty‐four commercially available, certified varieties of winter OSR, comprising eight OP, seven CMS hybrids and nine GMS hybrids, were grown under standardized conditions in an insect‐free glasshouse at Rothamsted Research in Hertfordshire, UK (51°48′34″ N, 0°21′23″ W; Table 1). Twenty‐three varieties were included on the 2013–2014 Recommended List for growers in England and Wales (AHDB, 2012), while one (SY Fighter) was a candidate for inclusion on the list.

**Table 1:** Oilseed rape varieties by breeding systems used in the trial

Open pollinated	GMS hybrid	CMS hybrid
Cash	Avatar	DK Excalibur
DK Cabernet	Compass	DK Expower
DK Camelot	Cracker	Flash
Fashion	Dimension	PR46W21
Quartz	Marathon	PT211
Rivalda	Rhino	DK Sequoia
Sesame	Thorin	PR45D05
Vision	Troy	
	SY Fighter	

In March 2013, seeds of all varieties were sown in trays containing a standard compost mix, comprising 75% peat, 12% sterilized loam, 10% lime‐free 5 mm grit and 3% medium‐grade vermiculite. The compost was fertilized with 16‐9‐12 NPK +2MgO at 3.5 kg m^−3^ (Osmocote Exact Mini 3–4, Scotts, UK). Seedlings were vernalized at the 3–4 leaf stage for 8 weeks at 5 °C, and seven plants of each variety were individually re‐potted to 21 cm diameter (4 l) pots, containing fresh standard compost mix. The potted plants were then evenly arranged in a randomized complete block (RCB) design, with seven blocks, in a glasshouse at a mean density of 8.5 pots m^−2^ (Fig. S1). An automated system watered plants twice daily, while supplementary lighting and heating were provided to ensure irradiance of at least 100 μmol m^−2^ s^−1^ from 05:00 to 21:00 and temperatures of at least 18 °C during the day, and 14 °C at night. The use of yellow sticky traps (Silvandersson, LBS Horticultural Supplies, Colne, UK) and predatory mites, *Amblyseius cucumeris* (Bioline AgroSciences, Little Clacton, UK), in addition to daily plant inspections, ensured the plants were pest‐free.

### Nectar collection and analysis

As our aim was to quantify inherent nectar production in OSR varieties and breeding systems, rather than assessing temporal availability of nectar, we measured 24‐h secretion rate rather than standing crop (Corbet, 2003). To control for flower age‐related differences in nectar production (Mohr & Jay, 1990; Mesquida *et al*., 1991; Pierre *et al*., 1996), nectar was sampled from flowers of the same age. Plants were inspected daily, in June, to record the day on which each began to flower. On each day, petals of all open flowers were marked with a permanent ink pen to ensure these older flowers were not used for nectar sampling. The plants were visited 24 h later, and the nectar was carefully removed from any flowers that had opened since the previous day by draining the inner nectaries using microcapillary tubes (5 μL, Drummond, Broomall, PA, USA). As outer (median) nectaries only secrete c. 5% nectar due to reduced phloem vascularization (Davis *et al*., 1994), nectar production was quantified from the inner (lateral) nectaries only. Nectar was then allowed to accumulate in these flowers for 24 h, prior to being sampled to measure 24‐h secretion rate. To control for potential diel patterns of nectar production within flowers (Pernal & Currie, 1998), all plants that were flowering within a block were sampled during a defined, 1‐h period (Table S1). As plants started flowering on different days, nectar samples were collected over multiple days (Table S2). One sample of nectar per plant comprised nectar collected from all flowers that had been drained 24 h previously (mean: 4.4 flowers, Table S2). Microcapillary tubes were immediately stored in 1.5 mL Eppendorf tubes placed on ice inside a cool box before being transferred to a freezer set at −20 °C. One variety, DK Sequoia, had not begun flowering by the time nectar sampling of the other varieties had commenced, and consequently, its nectar was not collected for analysis, although flower size and flower number were measured in this variety.

The 24‐h secretion rate of nectar collected was determined by dividing the length of the column of nectar in the microcapillary tube by the number of flowers sampled, to give mean volume (μL) per flower. High‐performance liquid chromatography (HPLC) was used to assess the concentration (μg μL^−1^) and composition of sugars in the nectar. Samples were diluted to 1 : 2000 with water (HPLC grade, Fisher Scientific, Loughborough, UK). A 10 μL volume of the diluted sample was introduced into the stream of 10 mm NaOH (flow rate 1 mL min^−1^) with an autosampler (ICS‐5000; Dionex, Thermo Fisher Scientific, Waltham, MA, USA) and passed through a CarboPac PA100 column (Dionex, Thermo Fisher Scientific, Waltham, MA, USA) to separate the sugars. Sugars were then detected with an electrochemical detector (ED40, Dionex, Thermo Fisher Scientific, Waltham, MA, USA), and chromeleon software (Thermo Fisher Scientific, Waltham, MA, USA) was used to determine concentration of the sugars by reference to calibrations using standards at 10 ppm. Sugar mass (μg) per flower was then calculated as the product of the volume of nectar per flower and sugar concentration.

### Flower size

Petal area was used as a surrogate measure of flower size. During the third week of flowering, one flower from the main raceme of each plant was selected when the petal laminae were perpendicular to the style and petals (mean: 2.0, Table S3) were carefully removed from the flower. The petals were affixed to transparency film with clear tape, scanned at 600 dpi, and the petal areas were calculated using imagej Version 1.44 (Schindelin *et al*., 2015).

### Number of flowers per plant

The mean number of flowers produced per plant was assessed for all 24 varieties once flowering had ceased (after c. 4 weeks; five blocks only) by summing the number of seed pods and flower‐abscission scars (on average 4.5 plants sampled per variety, Table S4).

### Statistical analyses

The mean secretion of nectar per flower in 24 h, expressed as total sugar mass, volume, total sugar concentration and fructose/glucose ratio, for all varieties except DK Sequoia, was compared among varieties using a linear mixed model (LMM) fitted using restricted maximum likelihood (REML), with block and sample date as the (crossed) random factors, to allow for environmental differences among sampling dates as well as differences associated with plant location in the glasshouse. The observed significance levels for comparing within breeding systems subsequently depended on the order of fitting of these nested terms, due to the resulting non‐orthogonal random structure because not all varieties and not all plants were sampled per day due to lack of flowering (Table S2). The range of observed probabilities over the six orders of term fitting is therefore presented, and denominator degrees of freedom (df) may be non‐integer.

Petal area and the total number of flowers produced per plant (all 24 varieties) were analysed using multi‐stratum anova to account for the RCB design. The estimate of nectar secreted over 24 h by all flowers produced per plant, for all varieties except Sequoia, was calculated as the product of nectar secretion per flower and number of flowers per plant, and was similarly analysed using anova.

In all the above analyses, fixed variety effects were partitioned to compare among and within the three breeding systems. Prior to analysis, nectar volumes were square‐root‐transformed, and the sugar mass per flower, number of flowers per plant and total nectar produced per plant were log‐transformed (base 10) to meet the assumptions of the analysis. Overall observed means are reported on the scale of analysis accompanied by ±SEM (with back‐transformed mean given in parentheses as appropriate).

We assessed the relationships between both nectar volume (log scale, base 10) and sugar mass (square root scale), respectively, and variety, flower size and number of flowers per plant using multiple regression with groups fitted as a LMM allowing for the RCB design in the random model. Neither interaction with variety was statistically significant for either response (Tables S5 and S6), so here we report *F*‐tests for dropping the explanatory variable of interest from the main effects models only. All analyses were done using genstat 18 (VSNi, 2015).

## Results

### Nectar production

**Figure 1:**
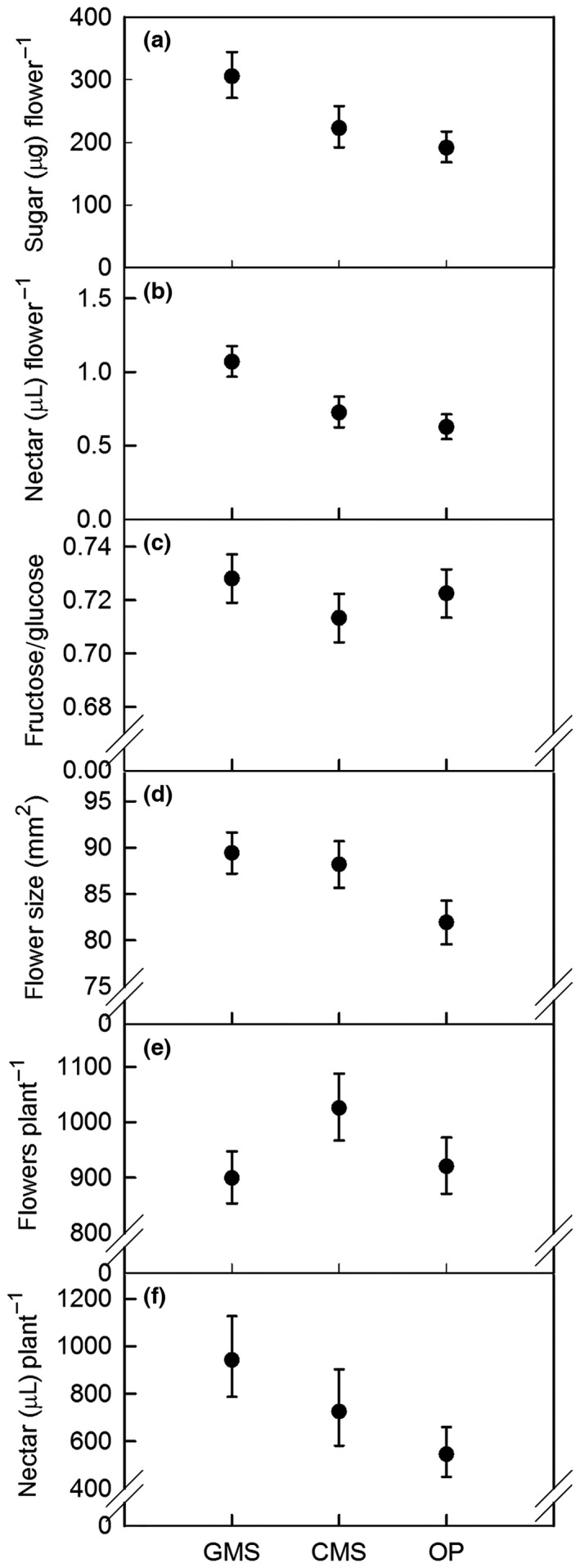
Mean metrics of oilseed rape floral traits, by breeding system (average *n *=* *54). GMS, genic male sterility hybrids; CMS, cytoplasmic male sterility hybrids; OP, open‐pollinated. Panels a–d are metrics per flower, while panels e and f are per plant. Error bars show 95% confidence intervals; data in all panels, except c and d, are back‐transformed to the natural scale.

**Figure 2:**
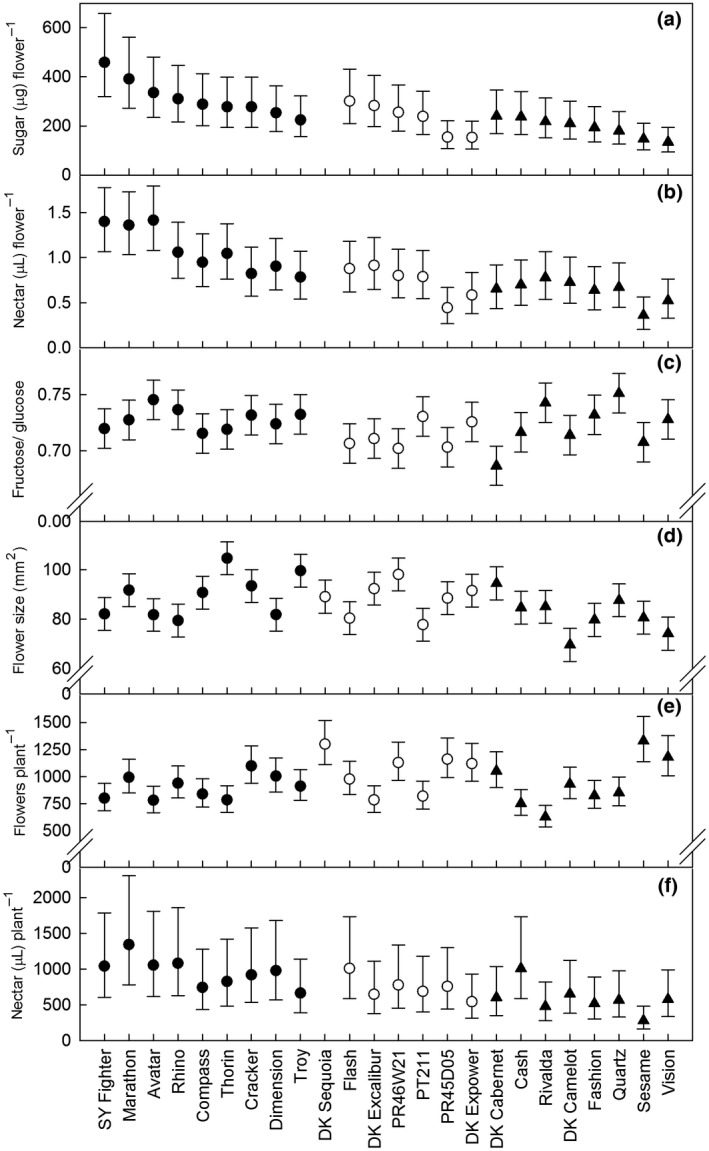
Mean metrics of oilseed rape floral traits by variety (*n *=* *7). Panels a–d are metrics per flower, while panels e and f are per plant. Closed circles: genic male sterility (GMS) hybrids; open circles: cytoplasmic male sterility (CMS) hybrids; closed triangles: open‐pollinated (OP). Error bars show 95% confidence intervals; data in all panels, except c and d, are back‐transformed to the natural scale.

Nectar was secreted by flowers of all 23 varieties included in this analysis. Across these varieties, flowers produced nectar with a mean of 2.38 (geometric mean 241.7 μg sugar) ± 0.020 (*N *=* *146) in 24 h. Per flower sugar mass differed among breeding systems (*F*_2,78.3_ **=* *14.60, *P *<* *0.001; Fig. 1a), with more produced by GMS hybrid varieties than by the CMS hybrid and OP varieties. There were no differences in the mass of sugar per flower within any of the breeding systems (within OP varieties – *P*: 0.259–0.442, df* *=* *7, 91.3; within CMS hybrid varieties – *P*: 0.071–0.114, df = 5, 78.7; within GMS hybrid varieties – *P*: 0.256–0.365, df = 8, 84.7; Fig. 2a).

The mean volume of nectar secreted by the inner nectaries per flower across all OSR varieties over 24 h was 0.90 (0.82 μL) ± 0.021 (*N *=* *150). Nectar volumes secreted by the three breeding systems differed (*F*_2,97.9_ **=* *15.03, *P *<* *0.001; Figure 1b), with the greatest volumes produced by GMS hybrid varieties. Nectar volumes did not differ within any of the breeding systems (within OP varieties – *P*: 0.202–0.289, df = 7, 107.6; within CMS hybrid varieties – *P*: 0.101–0.134, df = 5, 97.8; within GMS hybrid varieties – *P*: 0.054–0.065, df = 8, 104.5; Fig. 2b).

The mean concentration of nectar sugars across all plants was 324 ± 6.6 μg μL^−1^ (32.4% w/w; *N *=* *148), and differences were not found among (*F*_2,92.8_ **=* *0.20, *P *=* *0.818) or within any of the breeding systems (within OP varieties – *P*: 0.132–0.173, df = 7, 101.6; within CMS hybrid varieties – *P*: 0.127–0.183, df = 5, 93.5; within GMS hybrid varieties – *P*: 0.120–0.159, df = 8, 93.9). The majority of the sugar detected in OSR nectar was glucose (57.7% by mass), followed by fructose (41.7%) and sucrose (0.7%). The ratio of nectar fructose/glucose differed among the three breeding systems (*F*_2,94.5_ **=* *5.56, *P *=* *0.005; Fig. 1c), as well as within the OP varieties (all *P *<* *0.001, df = 7, 103.7). The ratios of nectar fructose/glucose within GMS and CMS hybrids were similar (*P*: 0.049–0.146, df: 8, 96.2; *P*: 0.046–0.061, df: 5, 95.3, respectively; Fig. 2c). The ratios of fructose/glucose tended to be greater in the GMS hybrids and lower in the CMS hybrids, while values for the OP varieties were spread over a wider range.

### Flower size

The mean area of petals from all 24 varieties was 86.57 ± 1.29 mm^2^ (*N *=* *160). There was a difference in petal size among the three breeding systems (*F*_2,130_ **=* *11.68, *P *<* *0.001; Fig. 1d). This indicates that flowers of CMS and GMS hybrid varieties tended to be larger than those of OP varieties. However, differences in petal size were also found among the varieties within each breeding system (within OP varieties: *F*_7,130_ **=* *5.38, *P *<* *0.001; within CMS hybrid varieties: *F*_6,130_ **=* *4.38, *P *<* *0.001; within GMS hybrid varieties: *F*_8,130_ **=* *6.94, *P *<* *0.001; Fig. 2d). There was no effect of flower size on either the volume of nectar produced per flower or sugar mass per flower (*F*_1,64.5_ **=* *2.14, *P *=* *0.149; *F*_1,54.6_ **=* *0.84, *P *=* *0.365, respectively; Tables S5 and S6).

### Number of flowers per plant

Plants produced a mean (*N *=* *109) of 2.97 (geometric mean 930.3 flowers) ±0.010. A difference was found in the number of flowers per plant among the three breeding systems (*F*_2,81_ **=* *6.08, *P *=* *0.003; Fig. 1e), where CMS hybrid varieties tended to produce more flowers per plant than the other breeding systems. There were also differences in flower production among the varieties within each breeding system (within OP varieties: *F*_7,81_ **=* *9.76, *P *<* *0.001; within CMS hybrid varieties: *F*_6,81_ **=* *5.77, *P *<* *0.001; within GMS hybrid varieties: *F*_8,81_ **=* *2.47, *P *<* *0.019; Fig. 2e). There was no effect of the number of flowers produced per plant on either the volume of nectar produced per flower or sugar mass per flower (*F*_1,66.5_ **=* *0.22, *P *=* *0.638; *F*_1,66.8_ **=* *0.89, *P *=* *0.348, respectively; Tables S5 and S6).

### Nectar resource per plant

The estimated mean volume (*N *=* *100) of nectar secreted over 24 h by all flowers that were produced per plant was 2.86 (geometric mean 728.1 μL) ± 0.030 and was found to differ among breeding systems (*F*_2,73_ **=* *8.52, *P *<* *0.001; Fig 1f), where nectar secreted by GMS varieties was greater than for OP varieties. There were no differences in the estimated secretion within the three breeding systems (within OP varieties: *F*_7,__73_ **=* *1.4, *P *=* *0.219; within CMS varieties: *F*_5,__73_ **=* *0.58, *P *=* *0.717; within GMS varieties: *F*_8,__73_ **=* *0.61, *P *=* *0.767; Fig. 2f).

## Discussion

This study is the first to compare nectar production in OSR varieties created using the genic male sterility (GMS) system with those bred by other methods. While we expected to observe varietal differences in metrics of nectar production that have been reported elsewhere (Pernal & Currie, 1998), such differences between breeding systems were less likely (c.f. Pernal & Currie, 1998; Pierre *et al*., 1999). We found that GMS hybrid varieties tended to produce the greatest volumes of nectar and amounts of nectar sugar per flower and larger, but fewer flowers per plant, whereas OP varieties consistently produced the smallest nectar volumes and amounts of nectar sugar per flower. With the exception of the ratio of fructose/glucose, we also found expression of nectar traits within all the breeding systems to be consistent. When these results were scaled to 24‐h nectar secretion by all flowers produced by an individual plant, we found that GMS varieties were estimated to yield 1.73 times more nectar than OP varieties.

Although not tested here, and in the absence of any evidence we can find in the literature, it is possible that GMS hybrids produce more nectar per flower than OP varieties as a result of heterosis endowing plants with larger or more active nectaries. If so, these effects of heterosis appear to be suppressed in the CMS hybrid varieties. All CMS hybrid varieties tested here were created with the Ogura system, which uses a cytoplasmic element originally derived from radish (*Raphanus sativus* L.; Yamagishi & Bhat, 2014) and has been implicated in less well developed, less productive nectaries (Pelletier *et al*., 1987; Mesquida *et al*., 1991). The presence of the radish cytoplasm in the F1 hybrid offspring, even with male‐fertility restored, could therefore depress nectar production relative to hybrids, such as those with GMS. In the Ogura system, the gene from the male‐fertile parent that restores fertility to the hybrid offspring was also transferred from *R*. *sativus* and includes unknown amounts of linked genes that may also influence the development and function of nectaries (Delourme *et al*., 1991; Bellaoui *et al*., 1999). When they compared phloem sap with nectar composition in field‐grown varieties of OSR, Bertazzini & Forlani (2016) concluded that the inter‐varietal variation observed in nectar production and composition, but not in phloem sap, was likely due to genotypic differences in nectary function. Of course, it is possible that apparent breeding system differences in nectar production may have been a result of inherent characteristics of parent lines that may have been shared within the breeding systems. Information on parent lines used in breeding trials is confidential to plant breeders, and so we are unable to explore possible confounding effects of shared parent lines; however, our work highlights that it would be beneficial for plant breeders and scientists to build on their existing collaborative platform.

The total concentration of sugars in nectar did not vary with variety or breeding system in this study, and thus, nectar sugar mass was directly related to nectar volume. Other studies have also found consistency in nectar sugar concentration between different varieties of OSR (Mohr & Jay, 1990; Mesquida *et al*., 1991; Pierre *et al*., 1996). While it is known that the family of SWEET protein transporters in plants, including brassicas, facilitate the diffusion of sucrose from photosynthetic to heterotrophic cells, such as nectaries, down a concentration gradient (Chen *et al*., 2012; Chen, 2014; Lin *et al*., 2014), little is known about the regulation of concentration in nectar sugar, and this is an emerging area of research.

Sucrose transported to the nectaries is hydrolysed to produce glucose and fructose (Lin *et al*., 2014), and all three sugars are secreted in nectar, in proportions that vary between species, within species and can be affected by soil conditions (Baude *et al*., 2011; Gijbels *et al*., 2014). We have shown that for varieties grown under similar soil conditions, the ratio of fructose/glucose varied at the genotypic level. Although yet to be confirmed, it has been hypothesized that secreted invertases at the nectaries may be involved in determining the final ratio of sucrose, fructose and glucose in brassicas (Lin *et al*., 2014). Among beekeepers, OSR honey is notorious for its rapid granulation (Calder, 1986), and a ratio of fructose to glucose below 1.11 in honey indicates a tendency to crystallize rapidly (Smanalieva & Senge, 2009). The ratio of these sugars in the nectar of older varieties of spring OSR has been shown to vary, but consistently recorded at ratios below 1.11 (Kevan *et al*., 1991). We found differences in the ratios of sugars within contemporary OP varieties and among breeding systems of winter OSR, which consistently indicated that all varieties we tested are likely to produce rapidly granulating honey.

In addition to intra‐ and inter‐breeding system differences in nectar production properties, we also found differences in flowering traits. Flowers of both CMS and GMS hybrid varieties were larger than those of OP varieties, suggesting that, contrary to observations in a confamilial, *Arabidopsis* (Miller *et al*., 2012), heterosis for flower size occurs in OSR. These differences in flower size are likely to be conserved in field conditions, because Cresswell *et al*. (2001) found that flower size of a single variety of OSR was consistent when plants were grown under a range of conditions. It is likely, therefore, that individual plants with larger flowers are more visually attractive to flower visitors, but it is not clear whether any effect would hold at the field scale. We found evidence of heterosis in flower production in CMS hybrids only, but the mechanism behind this is unclear and requires further investigation, because it has been shown that flower production in *R*. *sativus* plants with the CMS gene is lower than in plants without the CMS gene (Miyake *et al*., 2009).

Although Davis *et al*. (1996) demonstrated a clear relation between flower size and nectar volume in the closely related *B*. *rapa*, we found that neither flower size, nor the number of flowers produced per plant determines the volume of nectar or sugar mass produced per flower in *B*. *napus*. This suggests a lack of trade‐off between flower and nectar production and that size of floral display in *B*. *napus* may not represent an honest signal of nectar status for pollinators (Knauer & Schiestl, 2015). The lack of relation between floral size and nectar production also supports the hypothesis that floral nectar production is subject to complex gene expression and regulation (Liu & Thornburg, 2012; Lin *et al*., 2014).

While we recognize the composition of the UK Recommended List of OSR varieties (AHDB, 2012) is subject to change as new varieties are certified, the varieties tested here remain representative of the conventional breeding systems used in OSR plant‐breeding programmes. Of course, our observations of the inherent flowering and nectar properties of varieties and breeding systems tested may not be replicated in field‐grown plants. For example, fluctuations in nectar concentration occur with weather conditions (Farkas, 2008), time of day (Mohr & Jay, 1990) and over the course of the flowering season (Pierre *et al*., 1999). We also note that the mean number of flowers per plant in the present study was over twice that estimated in OSR grown in the field (Nedic *et al*., 2013) and may be in response to lack of insect visitors that are known to reduce flower production (Mesquida *et al*., 1988a), low planting density (Cresswell *et al*., 2001), or reduced stress in competing for nutrients and water than those grown in field conditions. Without accounting for the preceding caveats, our work suggests that OP and CMS hybrid plants would provide, on average, 57.9% and 76.9%, respectively, of the nectar resource per plant provided by GMS hybrid varieties (Fig. 1f).

In conclusion, we have shown that floral rewards in OSR varieties differ between breeding systems, and nectar production is functionally independent of flower production. We suggest that OSR varieties produced by the GMS hybrid technique could provide comparatively greater nectar rewards for insects that use the crop as a source of nutrition. We recommend research to understand and control the mechanisms of nectar production in OSR breeding systems and that the resource provided by different varieties of OSR to pollinators could be acknowledged in recommending varieties to growers. This work shows that plant breeding could be a useful tool in the quest for sustainable agricultural intensification: the implications of breeding system differences in rewards for both crop yield and insect pollinators, and also on honey production should be tested in the field, and scaled to the landscape and colony level using modelling approaches, such as the BEEHAVE model (Becher *et al*., 2014).

## Supporting information


**Figure S1.** Arrangement and layout of oilseed rape plants in the glasshouse.

**Table S1.** One‐hour sampling periods for plants in experimental blocks.

**Table S2.** Nectar and nectar sugar production per flower in 23 varieties of winter oilseed rape.

**Table S3.** Flower sizes of 24 varieties of winter oilseed rape

**Table S4.** Flower production in 24 varieties of winter oilseed rape.

**Table S5.** Relationships between nectar volume per flower and floral traits.

**Table S6.** Relationships between sugar mass per flower and floral traits.
