# Supplementary material for: Oilseed rape (Brassica napus) as a resource for farmland insect pollinators: quantifying floral traits in conventional varieties and breeding systems
Source: Glob Change Biol Bioenergy. 2017 Mar 10;9(8):1370–9. doi: 10.1111/gcbb.12438 (PMC5518758; doi:10.1111/gcbb.12438)
Supplement: Supplementary file 1 — Figure S1. Arrangement and layout of oilseed rape plants in the glasshouse. [file GCBB-9-1370-s001.docx]

**Carruthers et al. Supporting Information Fig. S1**

Arrangement and layout of oilseed rape plants in the glasshouse.


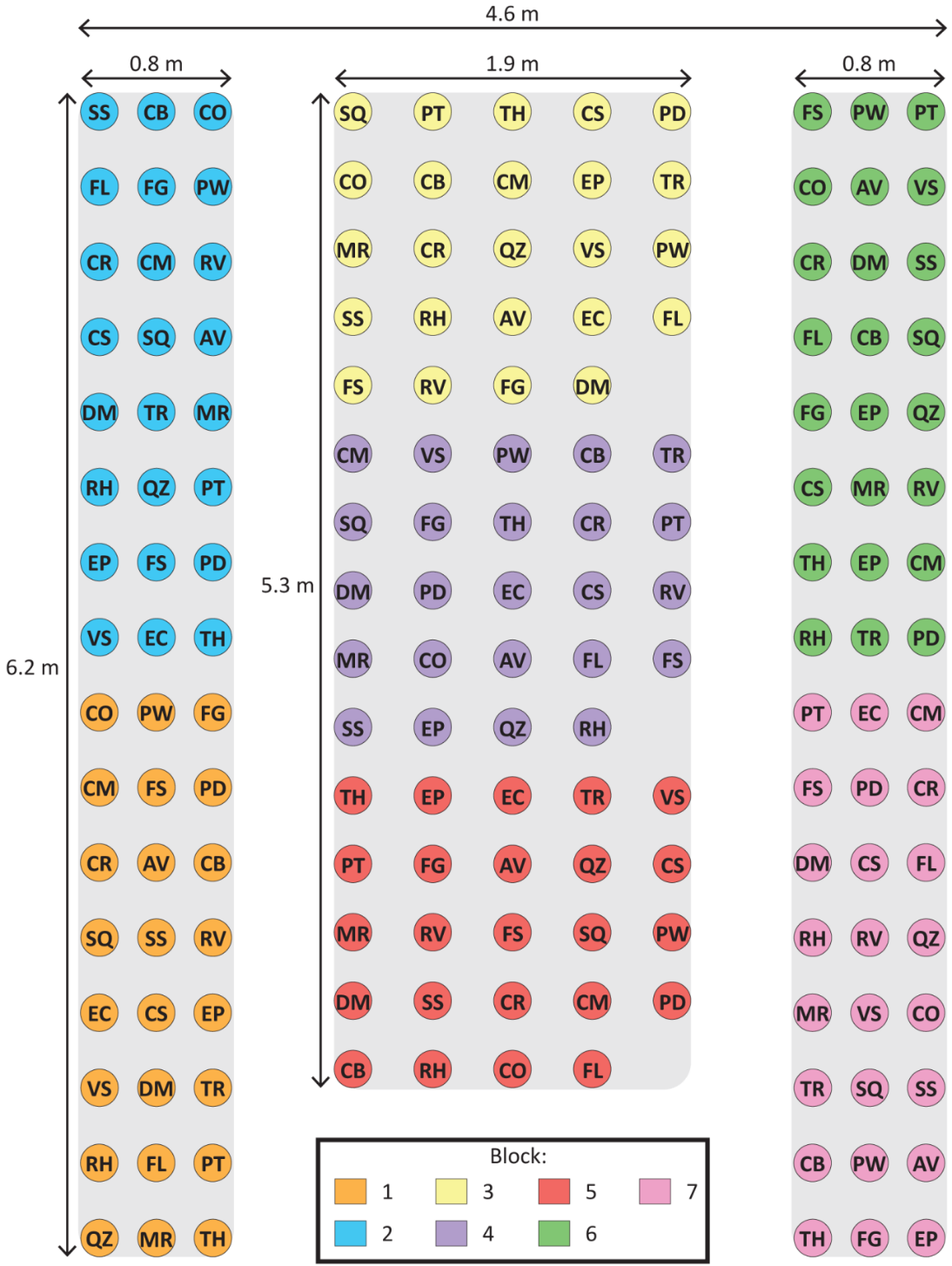


| AV | Avatar | DM | Dimension | MR | Marathon | RV | Rivalda |
| --- | --- | --- | --- | --- | --- | --- | --- |
| CB | DK Cabernet | EC | DK Excalibur | PD | PR45D05 | SQ | DK Sequoia |
| CM | DK Camelot | EP | DK Expower | PT | PT-211 | SS | Sesame |
| CO | Compass | FG | SY Fighter | PW | PR46W21 | TH | Thorin |
| CR | Cracker | FL | Flash | QZ | Quartz | TR | Troy |
| CS | Cash | FS | Fashion | RH | Rhino | VS | Vision |
