# Supplementary material for: Oilseed rape (Brassica napus) as a resource for farmland insect pollinators: quantifying floral traits in conventional varieties and breeding systems
Source: Glob Change Biol Bioenergy. 2017 Mar 10;9(8):1370–9. doi: 10.1111/gcbb.12438 (PMC5518758; doi:10.1111/gcbb.12438)
Supplement: Supplementary file 7 — Table S6. Relationships between sugar mass per flower and floral traits. [file GCBB-9-1370-s007.docx]

**Carruthers et al., Supporting Information Table S6**. Relationships between sugar mass per flower (square root scale) and floral traits. Results from fitting linear mixed models (LMMs) using restricted maximum likelihood (REML). F-statistics for dropping selected terms from three nested fixed models are given. Using the nomenclature of Wilkinson & Rogers (1973) the models are: Model 1 - full multiple regression with groups model ((A+N)*V = A + N + V + A.V + N.V); Model 2 - main effects model (A + N + V); Model 3 – main effect of variety only (V). In each case the random model represents the randomized complete block design (i.e. block/plant). Terms marked with an asterisk cannot be dropped as they are marginal to higher-order terms in the model.

| Fixed Term | Model 1 | Model 2 | Model 3 |
| --- | --- | --- | --- |
| Petal area (A) | * | *F*_1,54.6_=0.84, *P*=0.365 | - |
| Number of flowers plant^-1^ (N) | * | *F*_1,66.8_=0.89, *P*=0.348 | - |
| Variety (V) | * | *F*_22,66.5_=3.41, *P*<0.001 | *F*_22,75.1_=3.18, *P*<0.001 |
| Petal area.Variety (A.V) | *F*_20,21.3_=0.75, *P*=0.734 | - | - |
| Number of flowers plant^-1^.Variety (N.V) | *F*_20,20.2_=0.77, *P*=0.714 | - | - |

**Reference**

Wilkinson, G.N. & Rogers, C.E. (1973). Symbolic description of factorial models for analysis of variance. *Applied Statistics*, **22**, 392-399.
